# A case of treatment-resistant erosive pustular dermatosis of the scalp

**DOI:** 10.1177/2050313X241307126

**Published:** 2025-01-02

**Authors:** Zahra Rehan, Tracey Brown-Maher

**Affiliations:** Memorial University of Newfoundland, St. John’s, NL, Canada

**Keywords:** General dermatology, medical dermatology, inflammatory dermatoses, erosive pustular dermatosis, refractory dermatoses

## Abstract

Erosive pustular dermatosis is a rare and chronic inflammatory condition of the scalp which can be mistaken for cutaneous malignancy, precancerous lesions, dermatitis or pyoderma gangrenosum. The recurrent and resistant characteristics of erosive pustular dermatosis of the scalp pose a challenge to successful management and remission of the condition. The purpose of this case report is to provide management options and treatment recommendations for refractory cases of erosive pustular dermatosis of the scalp. We report a case of erosive pustular dermatosis of the scalp that proved resistant to treatment, with various therapeutic modalities explored to address the persistent condition. In our patient, multiple treatment modalities included topical and oral corticosteroids, topical and oral antibiotics, topical vitamin D analogue, topical calcineurin inhibitor and oral isotretinoin. Based on our findings, refractory disease progression of erosive pustular dermatosis of the scalp may be more responsive to combination therapy rather than monotherapy.

## Introduction

Erosive pustular dermatosis of the scalp (EPDS) is an under-recognized inflammatory condition that presents with localized areas of pustules or crusts overlying eroded plaques or nodules and subsequent scarring alopecia.^1^ EPDS is a diagnosis of exclusion; the presentation can often be mistaken for cutaneous malignancy and clinical correlation is critical as histopathology is non-specific.^2^ On histology, characteristic findings include atrophic epidermis and a mixed dermal inflammatory infiltrate of lymphocytes, neutrophils and occasional foreign body giant cells.^3^ The mainstay treatments are corticosteroids, antibiotics, calcineurin inhibitors, nonsteroidal anti-inflammatory drugs, retinoids and photodynamic therapy.^4^ Herein, we report a patient with erosive pustular dermatosis, who has demonstrated resistance to several mainstay treatments typically used to treat EPDS.

## Case report

A 72-year-old male with a history of basal cell carcinoma (BCC), actinic keratoses (AK) and seborrheic keratoses presented to the clinic with persistent crusts and a new erosion with eschar on the left temporal region, with no oozing or discharge. The initial biopsy revealed a foreign body reaction. In addition to counselling on continuing topical mometasone furoate cream, he was started on Doxycycline 100 mg. Concurrently, he was treated for underlying AK with cryotherapy. After 3 months, antibiotics were discontinued because of minimal relief and the formation of new erosions and crusts. At his request, he was started on Colchicine but noticed no improvement in left temple erosions with eschar. Six months later, he was started on a 6-month course of oral isotretinoin; during this time, small erosions started drying up and healing underneath the overlying haemorrhagic crust. With no significant improvements, isotretinoin was discontinued, and the patient was started on topical Calcipotriene ointment in conjunction with Dapsone gel. Three months later, gradual improvement was noted as old crusts healed and filled in. Debridement of eschar was offered at several follow-up appointments but promptly refused. In October 2022, 18 months after his initial presentation, there were new lesions on the vertex scalp and on the right temple. At this time, he was started on prednisone and protopic ointment; these have been used as treatment modalities for EPDS in previous case reports.^5^ After improvement, the patient returned to a higher dose of isotretinoin (40 mg) and continued with topical Dapsone 5% gel. Once he reached a plateau, there was a second biopsy done which revealed a BCC. After the BCC was excised, he developed some other areas of eschar that settled with topical Dapsone. Recently, he developed some new areas on the vertex scalp and right temple.

## Discussion

Primarily, EPDS affects older men who have accumulated sun damage on a bald scalp; however, females are also impacted by this condition.^6^ EPDS is an uncommon condition and usually an overlooked disease. Firstly, the diagnosis is often missed; there is a higher incidence of other cutaneous diseases affecting the same area, such as actinic keratosis, BCC and squamous cell carcinoma.^3^ Secondly, there are non-specific clinical, dermoscopic and histopathologic findings. Clinically, EPDS features are consistent with chronic pustules, erosions and crust.^3^ On dermoscopy, there is an absence of follicular ostia, dilated vessels and perifollicular serous crusts. The histopathology is non-specific, including atrophic epidermis and chronic inflammation consisting of lymphocytes, neutrophils and occasional foreign body giant cells.^7^

The goals of therapy are to reduce inflammation, heal erosions and prevent the progression of scarring alopecia to minimize permanent hair loss.^1^ The first line of management is topical corticosteroids and immunomodulating topical therapy.^6^ For steroid-sparing agents, topical calcineurin inhibitors, topical calcipotriol and retinoids have been used. Other therapeutic agents that have achieved complete resolution include photodynamic therapy, topical antibiotics such as Dapsone, topical tacrolimus, laser and wound dressings.^8^

EPDS can be a chronic recurring disease and the therapy can range from weeks to months. In case of refractory disease progression, combination therapy is common, usually in conjunction with a topical corticosteroid.^4^ With refractory EPDS, there may be benefit in trialling topical calcipotriol, short courses of systemic steroids, Doxycycline, isotretinoin, acitretin or Dapsone.^1^ In our male patient, most of these therapies were tested before therapeutic relief was achieved. In Australia, extensive management guidelines have been established that consider factors such as skin atrophy, response rate to topical corticosteroids, recurrence and refractory disease.^7^ In cases of no response to therapy, a biopsy should be considered.
